# From hybridomas to a robust microalgal-based production platform: molecular design of a diatom secreting monoclonal antibodies directed against the Marburg virus nucleoprotein

**DOI:** 10.1186/s12934-017-0745-2

**Published:** 2017-07-27

**Authors:** Franziska Hempel, Michael Maurer, Björn Brockmann, Christian Mayer, Nadine Biedenkopf, Anne Kelterbaum, Stephan Becker, Uwe G. Maier

**Affiliations:** 1 LOEWE Zentrum für Synthetische Mikrobiologie (SYNMIKRO), Philipps-Universität Marburg, Hans-Meerwein-Str. 6, 35043 Marburg, Germany; 2 Department for Cell Biology, Philipps-Universität Marburg, Karl-von-Frisch-Str. 8, 35043 Marburg, Germany; 3 Institute of Virology, Philipps-Universität Marburg, Hans-Meerwein-Str. 2, 35043 Marburg, Germany; 4 Deutsches Zentrum für Infektionsforschung (DZIF), partner site Gießen-Marburg-Langen, Germany; 5 Center for Molecular Biology of the University of Heidelberg (ZMBH), DKFZ-ZMBH Alliance, Im Neuenheimer Feld 282, 69120 Heidelberg, Germany; 6 Department of Microbial Bioactive Compounds, Interfaculty Institute of Microbiology and Infection Medicine, University of Tuebingen, Auf der Morgenstelle 28/E8, 72076 Tuebingen, Germany

**Keywords:** Microalgae, Biotechnology, Antibody production, Expression systems

## Abstract

**Background:**

The ideal protein expression system should provide recombinant proteins in high quality and quantity involving low production costs only. However, especially for complex therapeutic proteins like monoclonal antibodies many challenges remain to meet this goal and up to now production of monoclonal antibodies is very costly and delicate. Particularly, emerging disease outbreaks like Ebola virus in Western Africa in 2014–2016 make it necessary to reevaluate existing production platforms and develop robust and cheap alternatives that are easy to handle.

**Results:**

In this study, we engineered the microalga *Phaeodactylum tricornutum* to produce monoclonal IgG antibodies against the nucleoprotein of Marburg virus, a close relative of Ebola virus causing severe hemorrhagic fever with high fatality rates in humans. Sequences for both chains of a mouse IgG antibody were retrieved from a murine hybridoma cell line and implemented in the microalgal system. Fully assembled antibodies were shown to be secreted by the alga and antibodies were proven to be functional in western blot, ELISA as well as IFA studies just like the original hybridoma produced IgG. Furthermore, synthetic variants with constant regions of a rabbit IgG and human IgG with optimized codon usage were produced and characterized.

**Conclusions:**

This study highlights the potential of microalgae as robust and low cost expression platform for monoclonal antibodies secreting IgG antibodies directly into the culture medium. Microalgae possess rapid growth rates, need basically only water, air and sunlight for cultivation and are very easy to handle.

**Electronic supplementary material:**

The online version of this article (doi:10.1186/s12934-017-0745-2) contains supplementary material, which is available to authorized users.

## Background

Antibodies are important tools in diagnostics as well as in therapy and monoclonal antibodies currently represent one of the best-selling classes of biologics with US sales reaching 20.3 billion dollar in 2011 [1]. At the moment monoclonal antibodies are produced mainly in mammalian cell lines e.g. Chinese hamster ovary (CHO) cells [2, 3]. However, mammalian cell culture is very expensive to propagate and always bears the risk that cultures get contaminated with human pathogens representing serious bottlenecks for large scale production [4, 5]. Hence, alternative and safe expression systems for recombinant antibodies and lower production costs are highly desirable.

Bacteria and yeast are robust high yield expression systems widely used in biotechnology and currently provide a platform for the production of small synthetic antibody variants like single chain antibodies and nanobodies, which are interesting for therapeutic applications due to improved tissue penetration [6, 7]. The production of complex eukaryotic molecules like assembled IgG antibodies, which require a sophisticated folding machinery as well as eukaryotic post translational modifications, remain more challenging [8, 9]. Only recently though, it was shown that high level expression of fully assembled IgG antibodies within the cytoplasma of engineered *E. coli* cells is feasible representing a break through for low cost production of aglycosylated antibodies [10]. In the late 1990s plants came into focus as solar-fueled expression systems and the concept of *molecular farming* gained interest for production of edible vaccines but also for potential low-cost production of antibodies [11–14]. Today there are different examples for high level transient expression of monoclonal antibodies in glycomodified plants [15]. A direct comparison of monoclonal antibodies produced in plants and CHO cells revealed that plant produced antibodies against Ebola virus can be as effective as antibodies from CHO cells in vitro as well as in therapeutic studies with animal models [16, 17]. Nevertheless, cell culture based approaches (with higher plant cells or moss) with the antibody being secreted into the culture medium might be transferred to industrial scale more easily [18–20]. Much progress on plant produced antibodies has also been made in terms of glyco-engineering providing strains with humanized glycosylation profiles producing antibodies with a very homogenous glycopattern that show enhanced ADCC (antibody-dependent cell-mediated cytotoxicity) compared to mammalian antibodies [17, 21, 22].

Microalgae are currently in the focus of biotechnology due to high lipid content interesting for sustainable biodiesel production [23–26]. Additionally, microalgae-based production of recombinant proteins is highly interesting because algae are like land plants fueled by photosynthesis, the cultivation costs are very low and biomass productivity is much higher than for plants. Microalgae combine advantages of different expression systems as they are like prokaryotic systems very easy to handle, possess high growth rates, are very robust and easily scalable [27–30]. Furthermore microalgae provide eukaryotic modifications like N-glycosylation, which is important for the production of many mammalian proteins and might in future be engineered towards human specific profiles [31–33]. Many algal species are regarded as safe, they are no hosts to known human pathogens and have been used as nutrition supplements for many decades making them interesting especially for the production of therapeutic proteins [28, 34]. In 2003, a large single chain antibody was the first antibody produced in the plastid of *Chlamydomonas reinhardtii* [35] and 6 years later it was demonstrated that also a functional IgG antibody can be produced in the plastid of that model alga [36]. However, the production level was rather low, antibodies had to be extracted from the cells and lacked eukaryotic modifications which are not provided in chloroplasts. In the following years also synthetic antibodies like single chain immunotoxins and variants like camelid antibodies have been produced in the chloroplast of *C. reinhardtii*, which might provide several advantages over bacterial expression [37–39]. In 2011 it was shown in the diatom *Phaeodactylum tricornutum* that a human IgG antibody against the Hepatitis B Virus surface protein can be expressed very efficiently from the nuclear genome reaching production levels of ~9% of total soluble protein [40]. Further analyses revealed that the human IgG antibody can also be secreted into the culture medium [41]. The antibody was fully assembled, functional and without further purification steps already very pure as the alga seems to secret barely other proteins demonstrating the great potential of using microalgae as expression system for monoclonal IgG antibodies. A biochemical characterization of the *P. tricornutum* produced antibodies demonstrated that the immunoglobulins possess homogeneous C-terminal ends without proteolysis and carry high mannose-type *N*-glycans, which might be engineered towards human specific glycopatterns in future [33].

In this study, we implemented our previously established diatom system for generating monoclonal antibodies against the highly pathogenic Marburg virus (MARV). MARV belongs together with Ebola virus to the virus family *Filoviridae* which are the causative agents for severe hemorrhagic fevers with high fatality rates in humans. Filoviruses are endemic in Africa and their potential to threaten private and public health is high, as it was seen during the recent outbreak of Ebola virus in West-Africa (2014–2016) with more than 28,000 infections and more than 11,000 fatalities. So far, neither for Ebola virus nor for MARV licensed vaccines or treatments for human use are available. MARV particles consist of seven viral proteins. One of the main components of the viral transcription and replication complex is the nucleoprotein (NP) which encapsidates the viral genomic RNA. Although NP is a major target of antibodies in the infected mammalian host their function is currently enigmatic [42]. In order to implement the diatom system for the generation of a MARV-specific monoclonal antibody, we used a previously generated mouse monoclonal antibody against the MARV NP. We retrieved sequences for the light and heavy chain of the murine IgG antibody against the NP directly from the respective hybridoma cell line, expressed the antibody in the microalga *P. tricornutum* and compared its functionality directly to the original hybridoma produced antibody. The antibody is secreted by the diatom as completely assembled molecule into the algal culture medium and was characterized and tested in different applications such as western blot assays, ELISA, as well as immunofluorescence confirming the high quality of the algal produced antibody. Besides the original IgG antibody two chimeric antibodies with rabbit and human Fc-regions, respectively, were generated and expressed in the alga. All variants were proven to be functional demonstrating the ease of using *P. tricornutum* as a low-cost expression system for IgG antibodies.

## Results

### Expression and secretion of fully assembled α MARV NP antibodies by *P. tricornutum*

Sequences for antibodies against the Marburg virus nucleoprotein (MARV NP) are not known so far. Hence, in a first step cDNA sequences encoding the light and heavy chain of an α MARV NP IgG antibody were retrieved from an existing hybridoma cell line. Nucleotide sequences were amplified via RT-PCR (GenBank: KY441466, KY441467) using a set of degenerate primers described previously by Wang et al. and Li et al. [43, 44]. Besides the full length sequences for the light and heavy chain, two aberrant forms were detected for the light chain. One sequence corresponded to the non-functional form κ-138 which is commonly found in hybridoma cell lines generated with SP2/0-Ag14 cells and traces back to the fusion partner [45], the second aberrant sequence was not reported so far (GenBank: FN422002.1, KY441468). Altogether, the full length sequences including the signal peptides were amplified and allowed a broad application spectrum e.g. the heterologous expression of the complete IgG antibody but also the production of synthetic variants like chimeric humanized IgG antibodies interesting for therapeutic applications.

**Fig. 1:**
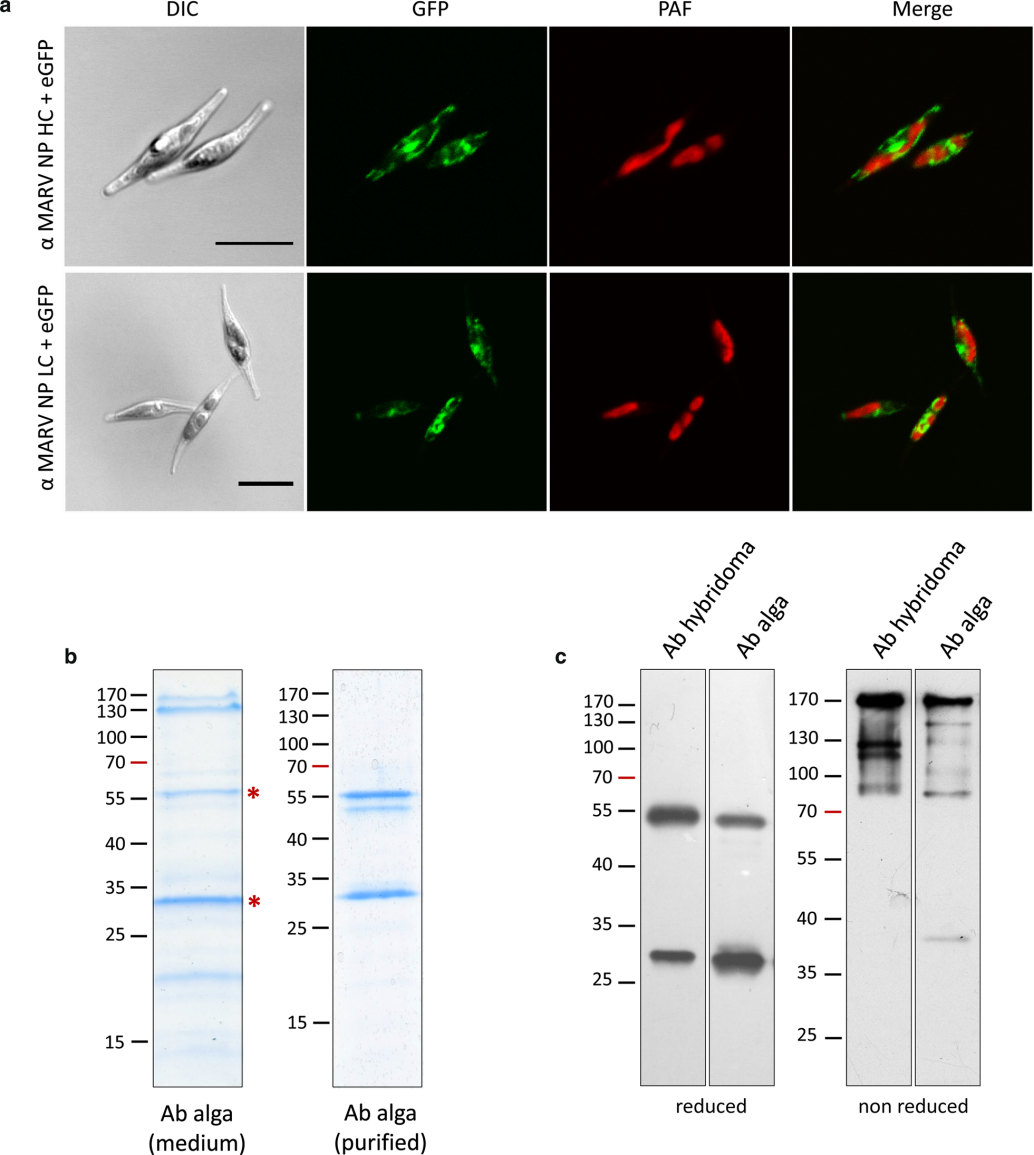
*Phaeodactylum tricornutum* secretes fully assembled α MARV NP IgG antibodies into the medium. **a** In vivo localization studies on the expression of heavy and light α MARV NP antibody chains in *P. tricornutum*. Sequences for the light and heavy chain of the murine IgG2a antibody against the Marburg virus nucleoprotein were retrieved from a hybridoma cell line and expressed as GFP fusion proteins in the diatom *P. tricornutum*. Both fusion proteins are expressed in the heterologous system without codon optimization and accumulate within the ER of the cells demonstrating that the murine signal peptide is sufficient for ER targeting. *Scale bar* represents 10 µm. **b** Purity analysis of secreted α MARV NP IgG antibodies. Proteins of 30 ml culture medium were concentrated with centrifugal filter units (cut off of 10 kDa), separated by SDS-PAGE and stained with Coomassie. Besides the antibody heavy and light chain (marked with *asterisks*) two dominant signals were detected at ~130 kDa representing a known phosphatase (Phatr2 ID: 47612, verified by mass spectrometry). After protein A purification no background remained, but a weak signal for a HC variant with reduced molecular weight is detected (verified by mass spectrometry). **c** Biochemical analyses of secreted α MARV NP IgG antibodies. 100 ng of purified hybridoma produced antibody and antibody from the algal medium were prepared under reduced and non reduced conditions, separated by SDS-PAGE and analysed in western blot studies using an α mouse secondary antibody for detection. Under reduced conditions heavy and light chain of the antibody are clearly separated and appear as distinct signals of 55 and 30 kDa. In case of the algal produced antibody the light chain seems to accumulate to slightly higher amounts. Under non reduced conditions disulfide bridges stay intact and the assembled antibody consisting of two heavy and two light chains is detected with a size of about 170 kDa. Lower molecular weight signals are observed in case of both antibodies with no significant difference. *Ab* antibody, *HC* heavy chain, *LC* light chain, *MARV* Marburg virus, *NP* nucleoprotein, *PAF* plastid autofluorescence

Initially, the sequences for heavy and light chain of the murine MARV NP antibody were expressed as eGFP fusion constructs in *P. tricornutum* to check whether expression of the antibody chains and their targeting to the endoplasmatic reticulum is supported by the heterologous expression system. For both fusion proteins a typical ER localization was observed (Fig. 1a). Subsequently, both sequences were expressed in one cell line without the eGFP fusion part under the control of the inducible nitrate reductase promoter of *P. tricornutum*. The antibody was expressed by the alga and secreted into the culture medium. For a first check on antibody quality, proteins of 30 ml medium were separated by SDS gel electrophoresis and stained with Coomassie demonstrating that the secreted antibody is already relatively pure (Fig. 1b). In the following, antibody functionality was tested in different assays and specificity was compared directly to the purified mouse hybridoma antibody.

### Algae vs. hybridoma produced antibody

**Fig. 2:**
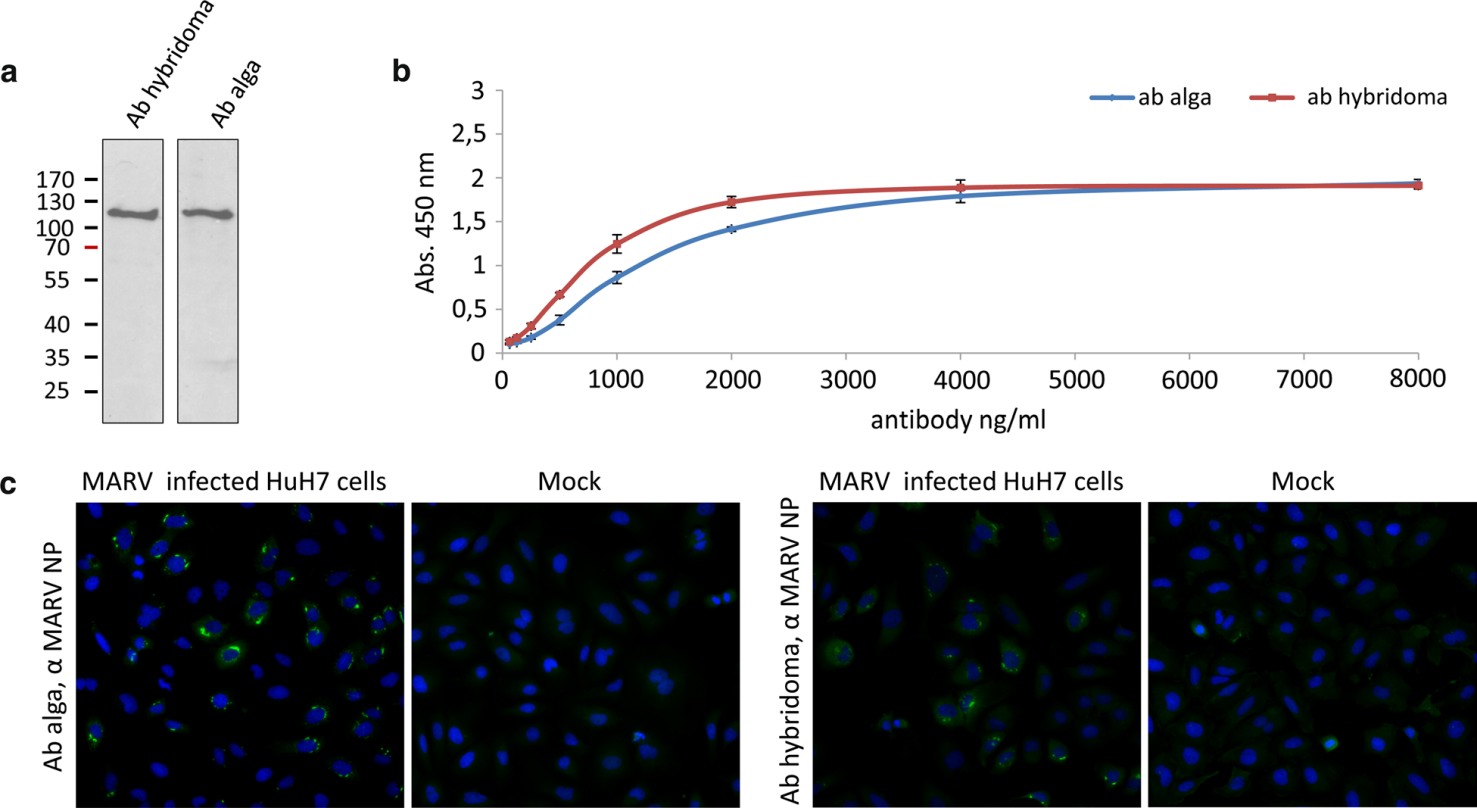
Comparing functionality of algae and hybridoma produced α MARV NP antibodies in different in vitro assays. **a** Western blot analyses demonstrate that algae and hybridoma produced antibodies recognize the MARV NP protein equally good. Cell extract of MARV infected HuH7 cells was separated by SDS-PAGE, followed by western blot analyses using 500 ng/ml antibody for detection. In case of both antibodies the nucleoprotein is detected with a clear and distinct signal at about 120 kDa. **b** ELISA studies measuring the binding affinity for MARV NP protein reveal that the algal produced antibody, which was retrieved directly from the culture medium is able to bind the native protein, but not as good as the hybridoma produced antibody. Medium of wild type cells and samples without NP protein served as negative controls. **c** Immunofluorescence assays with MARV infected HuH7 cells (20 h post infection) show for both antibodies a clear labeling for MARV NP, which accumulates in *dot* like structures (*green*) around the nucleus (*blue* DAPI stained). No labeling is observed for mock HuH7 cells. *Error bars* indicate standard deviation (n = 3)

Non-reducing SDS gel electrophoresis and western blot analyses demonstrated that the antibodies secreted by the alga are assembled in a high molecular weight complex of about 170 kDa corresponding to an IgG molecule consisting of two heavy and two light chains (Fig. 1c). Additionally, some lower molecular weight signals were detected, but no significant difference compared to the purified MARV NP antibody produced by the hybridoma cell line was observed. In further assays, the specificity of the antibody, i.e. its ability to bind to the target antigen, was tested in different in vitro assays. For testing the algal produced antibody in western blot analysis, HuH7 cells were infected with Marburg virus at a MOI of 1 and lysed 20 h post infection. Cell lysates were inactivated and aliquots were processed by SDS PAGE and western blotting. The hybridoma and the alga NP-specific antibodies were used to detect NP at a concentration of 500 ng/µl. Both antibodies showed a comparable sensitivity for NP (Fig. 2a). Furthermore, ELISA studies were performed to investigate whether the antibody is able to bind to native MARV NP, as well. For these studies 150 ng of recombinant MARV NP were coated to wells and antibody binding was tested. The assay demonstrated that the antibody secreted by the alga was also fully functional in ELISA (Fig. 2b). However, the binding efficiency was slightly lower compared to the purified hybridoma antibody. To exclude inhibitory effects of other proteins within the medium of the alga and to improve antibody quality, the algal produced antibody was purified with protein A agarose. Antibody purification had no significant effect on binding efficiency in ELISA (Additional file 1). However, the Coomassie staining of the purified antibody revealed a weak additional signal for a HC variant with slightly reduced molecular weight (Fig. 1b) (verified by mass spectrometry), which might be a reason for slightly impaired binding compared to the original hybridoma produced antibody. Finally, the algal antibody was tested in immunofluorescence analysis. Huh7 cells were infected with MARV (MOI 1) for 20 h. Cells were fixed, inactivated and stained with the algal produced and the original hybridoma antibody. Again, both antibodies clearly recognized the typical viral inclusion bodies which are formed during viral infection due to NP accumulation [46] (Fig. 2c).

### Expression of synthetic antibody variants

**Fig. 3:**
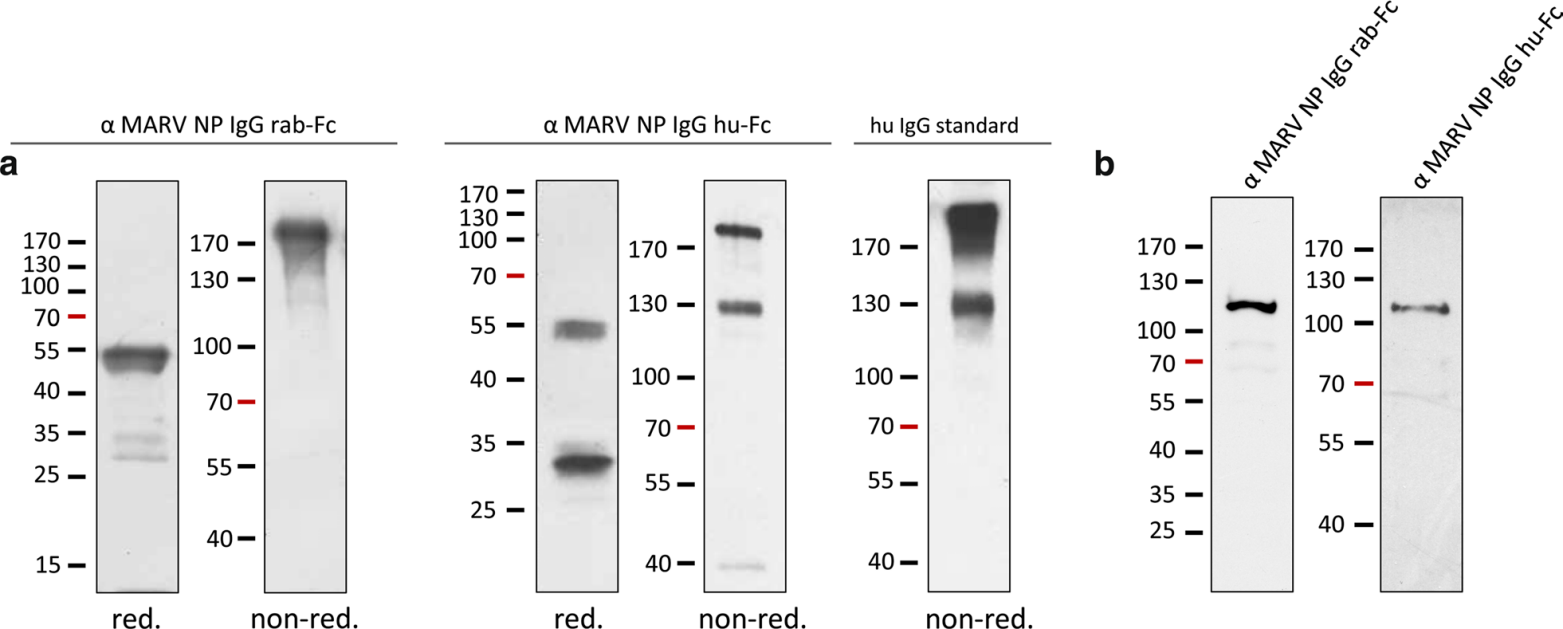
Production of chimeric α MARV NP IgG antibodies in *P. tricornutum*. **a** Chimeric antibody variants with rabbit and human Fc-regions are like the original murine IgG complex secreted by *P. tricornutum*. The secreted antibodies are assembled as demonstrated by gelelectrophoretic separation of reduced and non-reduced samples followed by western blot assays with α human and α rabbit secondary antibodies respectively (100 ng of algal produced antibody were loaded). Beside the ~170 kDa signal of the completely assembled IgG complex a second signal of about 130 kDa was detected for the humanized antibody version, however this is also true for a sample of human IgG standard. **b** Western blot assays using both chimeric variants as detection antibody for MARV NP demonstrate that both antibodies are functional and recognize the target antigen. Cell extract of MARV infected HuH7 cells was separated by SDS-PAGE, followed by western blot analyses using 500 ng/ml antibody for detection

On the basis of the cDNA sequences that were obtained for the light and heavy chain of the MARV-NP antibody and published sequences for other mammalian IgGs, chimeric antibody variants with either rabbit or human Fc-regions were generated and expressed in *P.* *tricornutum* (GenBank: KY441469, KY441470, KY441471, KY441472). Especially humanized antibodies are of course interesting for potential therapeutic applications. Sequences for the constant regions of rabbit and human IgG were synthesized (by Life Technologies) and fused to the variable regions via PCR or via cloning. In case of the humanized antibody the codon usage of the complete sequence was adapted to *P. tricornutum* codon usage. Assembly assays demonstrated that the chimeric IgG antibodies were correctly folded and secreted as a fully assembled complex (Fig. 3a). In case of the humanized version, the non-reduced sample showed beside the 170 kDa signal a distinct signal of approximately 130 kDa. This pattern was also observed when using a human IgG standard and might represent an intermediate complex being the result of fragmentation during sample preparation (Fig. 3a). The ability of the chimeric antibody variants to bind MARV-NP protein was confirmed in western blot assays using secondary antibodies against rabbit and human IgG, respectively (Fig. 3b). Quantification assays gave a yield of about 1300 ng/ml secreted antibody in case of the humanized version and approximately 500 ng/ml in case of the murine antibody.

## Discussion

Monoclonal antibodies are important instruments in immunotherapy of cancer, autoimmune diseases and Alzheimer disease and the production of monoclonal antibodies in CHO cells has become a multibillion dollar industry worldwide. However, high production costs and potential virus contamination represent critical drawbacks and hence alternative production platforms are required. Here, we implemented diatoms as a platform for the generation of a mouse monoclonal antibody against the highly pathogenic MARV. MARV is closely related to Ebola virus (family Filoviridae); both are endemic in Africa causing severe hemorrhagic fevers in humans and nonhuman primates. To date, there are neither licensed vaccines nor therapeutics available against Marburg or Ebola virus infections in humans. Therefore, containment of Marburg or Ebola virus outbreaks is relying on a rapid response mechanism in order to mitigate their consequences. Novel strategies to combat filovirus outbreaks include the large scale production and use of neutralizing monoclonal antibodies as a therapeutic tool. A recent example is a cocktail of monoclonal antibodies (ZMapp) against Ebola virus which was used as experimental therapy during the Ebola virus outbreak in West-Africa in 2014–2016 [47]. Furthermore, a single monoclonal antibody, isolated from a survivor of Ebola, was shown to protect nonhuman primates even 5 days post infection [48] demonstrating the great potential of antibody therapies not only for chronic diseases and cancer but also infectious disease. Additionally, single-domain antibodies were demonstrated as reliable and fast diagnostic tools as it has been shown for IgG and llama single domain antibodies against MARV NP [49, 50]. In this study, we tested our previously described diatom system for the rapid generation of a monoclonal mouse antibody against the nucleoprotein of Marburg virus.

We retrieved sequences for the heavy and light chain of an IgG2a antibody against MARV NP from a murine hybridoma cell line, which is to our knowledge the first published sequence of a MARV NP antibody. Subsequently, both sequences including the murine signal peptides were expressed within the microalga *P. tricornutum* resulting in completely assembled and functional IgG antibodies secreted into the medium. Functionality of the antibody was tested in WB and IFA assays demonstrating that the quality of the algal produced antibody is comparable to the original hybridoma produced antibody. In ELISA the algal produced antibody showed in comparison to the original hybridoma produced antibody reduced binding affinity, which might be due to partially inaccurate signal peptide cleavage (as it was observed for another IgG produced in *P. tricornutum* [33]). Indeed, the additional HC variant detected after protein A purification (Fig. 1b) might be a result of partially inaccurate processing of the signal peptide, a problem that might be overcome by using endogenous targeting signals in future. The expression level for this murine antibody was with 500 ng/ml medium lower than for the previously in *P. tricornutum* expressed human IgG [41], which reached expression levels of 2500 ng/ml [41]. However, the murine sequences that were retrieved from the hybridoma cell line were expressed without codon-optimization, which probably represents the main reason for lower expression levels. This was confirmed by using a chimeric immunoglobulin with the constant part of a human IgG antibody and full codon-optimization within the variable regions, which showed expression levels of about 1300 ng/ml. In this study, no further strategies for expression optimization were applied but preliminary studies indicate that for example multiple integration events of the plasmid can enhance expression levels. With regard to cell culture the cell density is certainly another way to optimize antibody production. In the present studies cells were grown in Erlenmeyer flasks with optical cell densities of approximately 2 (OD _600nm_) as a maximum, however large scale cultivation conditions e.g. using flat panel reactors with continuous feeding will result in much higher cell densities and potentially provide much higher antibody concentrations in the medium.

## Conclusions

This study highlights the ease and great potential of using microalgae as expression system for monoclonal antibodies. It took only a few weeks from sequence retrieval to the generation of microalgae secreting the antibody into the culture medium and as the antibodies within the salt water medium are already relatively pure, downstream processing is facilitated remarkably. Microalgae represent an attractive and robust production platform as they are easy to handle, provide rapid growth rates at low cost since they need basically only light, air and water. Furthermore, microalgae are no host for human pathogens, minimizing the risk for human pathogenic contaminations—a further important safety and cost factor for the production of therapeutic proteins. While microalgal antibody production can presently not compete with mammalian systems in terms of production efficiency, future bioengineering of algae will certainly improve expression levels, secretion performance and enable the humanization of glycosylation patterns. The dramatic Ebola outbreak in West Africa clearly demands robust and fast expression systems for therapeutic antibodies that can be handled easily and at low cost eventually even on-site, which might be feasible with a microalgal system in future.

## Methods

### Amplification of heavy and light antibody chains and plasmid construction

RNA of 0.5 × 10^6^ cells of hybridoma cell line MARV 59-9-10-7 (SP2/0-Ag14 + BALB/c), producing an IgG2a antibody against MARV NP protein (BioGenes GmbH), was isolated with the *RNeasy Plus Mini* Kit (Quiagen). cDNA was generated using 1 µg RNA, (dT)_18_ oligonucleotides and *Superscript II reverse transcriptase* according to manufacturer’s instructions. Amplification of the sequences for the heavy and the light antibody chain was performed with *Taq polymerase* according to [44]. A set of degenerate oligonucleotides binding in the 5′-region of the signal peptide [43] and oligonucleotides binding in the constant region of light and heavy chain [44] was tested for initial amplification of the variable region of both antibody chains. For final amplifications of the full-length sequences and the insertion of restriction sites the following oligonucleotides were used: 5HC_GCGGCCGCATGGGATGCAGCTGGGTAATGC, 3HC_CCGCGGTCATTTACCCGGAGTCCGGGAGAAG, 5LC_GAATTCATGGAGACAGACACACTCCTGC, 3LC_AAGCTTTTAACACTCATTCCTGTTGAAG. For expression of the complete antibody both sequences were cloned in the plasmid pPha-DUAL-[2 × NR] (Genbank: JN180664), which carries two multiple cloning sites both being under the control of *P. tricornutum* nitrate reductase promoter/terminator. For in vivo localization studies the sequences were fused with the sequence of eGFP and cloned into the plasmid pPha-NR (Genbank: JN180663). Chimeric sequences with rabbit and human Fc-regions were generated either by fusion-PCR or by introducing the restriction site BglII within the linker region and fusing variable and constant parts by cloning. Templates for the constant regions of human and rabbit IgGs were synthesized by Life Technologies.

### Antibody expression, harvesting and quantification

*Phaeodactylum tricornutum* (Bohlin, University of Texas Culture Collection, strain 646) was stably transfected by biolistic transfection as described previously [51] using M10 tungsten particles and 1350 psi rupture discs together with the particle delivery system Bio-Rad Biolistic PDS-1000/He. Cells were cultivated in f/2 medium under constant illumination (80 μmol photons/m^2^ s^1^) at 22 °C. In contrast to standard f/2 medium (based on the f medium of Guillard and Ryther 1962 with all supplements reduced by half) cells were grown with 1.6% tropic marine, without silicate and with 1.5 mM NH_4_Cl as nitrogen source; pH was adjusted to 7.4. Liquid cultures were grown with agitation (150 rpm) in a volume of 50–1000 ml. For transfection and long term storage cells were grown on solid f/2 plates supplemented with 1.3% agar and 75 µg/ml Zeocin. For antibody production cells were grown to an OD_600nm_ of 1.5 and antibody expression was induced by transferring cells to fresh f/2 medium containing 0.9 mM NaNO_3_ but lacking ammonia. After 2–3 days of antibody expression cells were removed by centrifugation (1500×*g*, 10 min) and the medium was filtered (⌀ 0.2 µm) [41]. Antibody within the medium was harvested by using centrifugal filter units (cut off of 10 kDa), buffered in PBS and used directly for functionality assays without further purification steps. For ELISA the antibody was either used directly or purified with protein A agarose (ThermoScientific) according to manufacturer’s instructions. In case of the original hybridoma produced MARV NP antibody used for comparative analyses a Protein A-purified stock solution was utilized. The antibody concentration was measured as described previously [41] using the *Easy Titer Mouse IgG Assay Kit*, *Easy Titer Rabbit IgG Assay Kit* and the *Easy Titer Human IgG (H* + *L) Assay Kit* (ThermoScientific) according to manufacture instructions with mouse, rabbit or human IgG as standard (500–15.6 ng/ml), respectively.

### Western blot and ELISA

Antibody quality was initially analysed via SDS-PAGE and western blot analysis. Under reduced conditions standard SDS Laemmli buffer was used and samples were incubated for 10 min at 96 °C. Under non reduced conditions no ß-mercaptoethanol but 20 mM NEM (*N*-ethylmaleinimid) was added to the Laemmli buffer and samples were incubated for 10 min at 70 °C according to [52].

The ability of the antibody to bind to Marburg Virus NP protein was analysed by ELISA and western blot analysis. For ELISA analyses 150 ng of recombinant NP protein was coated to the wells over night as described previously [40]. After blocking and subsequent washing steps the wells were incubated with serial dilutions of algal produced antibody as well as the original antibody produced in hybridoma cells. Antibody bound to MARV NP was detected with an anti-mouse IgG secondary antibody coupled to HRP (Sigma-Aldrich). Medium of wild type cells, PBS and samples without NP protein served as negative controls. All measurements were carried out in triplicates. For western blot analysis, HuH7 cells were infected with Marburg virus as described below and cell extract was separated by SDS-PAGE. 500 ng/ml algal produced antibody and original antibody were used for NP binding. Depending on the primary antibody anti-mouse, anti-rabbit and anti-human IgG secondary antibodies coupled to HRP (Sigma-Aldrich) were used for detection.

### Mammalian cell culture and MARV infection

HuH7 (human hepatoma) cells or Vero (african green monkey kidney) were cultivated with Dulbecco’s modified Eagle medium supplemented with penicillin and streptomycin, 5 mM glutamine and 10% fetal calf serum at 37 °C and 5% CO_2_.

Infections with Marburg virus were performed under highest safety standards in the biosafety level 4 laboratory at the Institute of Virology, Philipps University Marburg according to national safety regulations. Vero cells were infected with recombinant Marburg virus at a MOI of 0.1. Seven days post infection, cells were lysed by SDS treatment, boiled and lysates were used to perform SDS-PAGE and western blot analyses.

### Fluorescence microscopy

*Phaeodactylum tricornutum* cells were fixed with 4% PFA and 0.5% GA and localization of GFP fusion proteins was analysed with a confocal laser scanning microscope Leica TCS SP2 using a PL APO 63×/1.32–0.60, Oil Ph3 CS objective. GFP and chlorophyll were excited at 488 nm with an Ar 65 mW laser and fluorescence was detected at 500–520 and 680–720 nm, respectively. For immunofluorescence analyses, HuH7 cells on cover slips were infected with a MOI of 1. 20 h post infection, cells were fixed and virus was inactivated with 4% Paraformaldehyde for 16 h at 4 °C. Fresh 4% PFA was added to the cells before bringing them out of the BSL4 lab according to our standard operating protocols. After the additional incubation with 4% PFA for 16 h at 4 °C, cells were treated with 100 mM Glycine in PBS_def_ for blocking unspecific background binding and permeabilized with 0.1% Triton X100/PBS_def_. Cover slips were stored in Blocking Buffer [2% BSA, 5% glycerine, 0.005% natrium azide, 0.2% Tween 20 in PBS_def_] until staining for no more than 5 days. Primary antibodies were used in a concentration of 85 ng/µl.

## Additional file

**Additional file 1.** Comparing functionality of purified algal and hybridoma produced α MARV NP antibodies in ELISA. ELISA studies measuring the binding affinity for MARV NP protein revealed that the algal produced antibodies (protein A purified) bind to the protein, but affinity is reduced compared to the hybridoma produced antibodies. There was no significant difference in binding efficiency between antibodies taken directly from the algal medium (Fig. 2b) and purified algal antibodies. Error bars indicate standard deviation (n = 3).

## Abbreviations

HC: heavy chain
LC: light chain
MARV: Marburg virus
NP: nucleoprotein
