# Supplementary material for: From hybridomas to a robust microalgal-based production platform: molecular design of a diatom secreting monoclonal antibodies directed against the Marburg virus nucleoprotein
Source: Microb Cell Fact. 2017 Jul 27;16:131. doi: 10.1186/s12934-017-0745-2 (PMC5531009; doi:10.1186/s12934-017-0745-2)
Supplement: Supplementary file 1 — Additional file 1. Comparing functionality of purified algal and hybridoma produced α MARV NP antibodies in ELISA. ELISA studies measuring the binding affinity for MARV NP protein revealed that the algal produced antibodies (protein A purified) bind to the protein, but affinity is reduced compared to the hybridoma produced antibodies. There was no significant difference in binding efficiency between antibodies taken directly from the algal medium (Fig. 2b) and purified algal antibodies. Error bars indicate standard deviation (n = 3). [file 12934_2017_745_MOESM1_ESM.pdf]

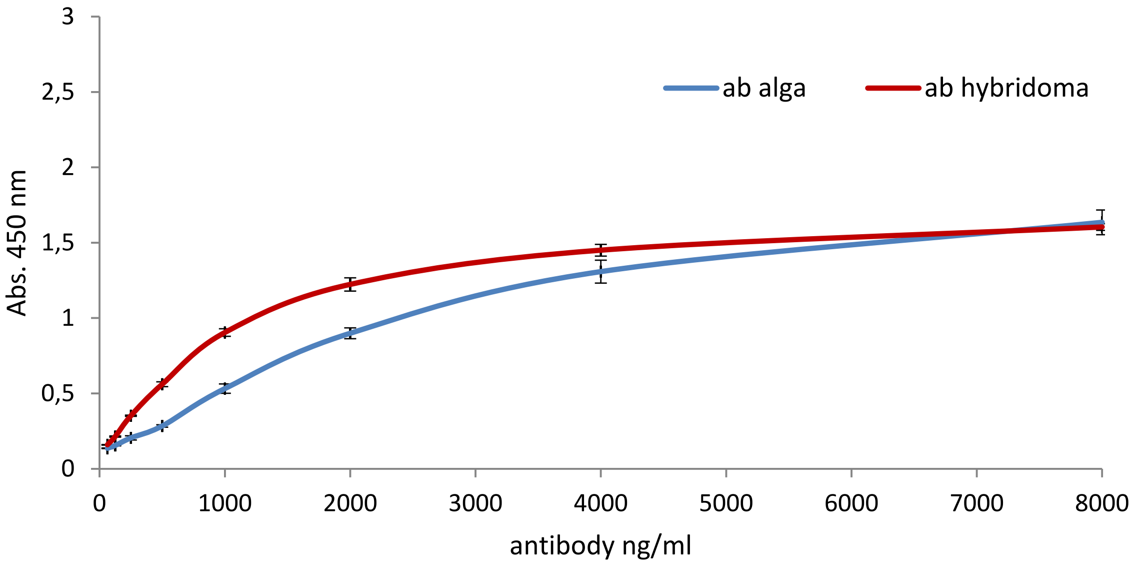

Additional file 1: Comparing functionality of purified algal and hybridoma produced α MARV NP antibodies in ELISA. ELISA studies measuring the binding affinity for MARV NP protein revealed that the algal produced antibodies (protein A purified) bind to the protein, but affinity is reduced compared to the hybridoma produced antibodies. There was no significant difference in binding efficiency between antibodies taken directly from the algal medium (Fig. 2b) and purified algal antibodies. Error bars indicate standard deviation (n=3).
